# Supplementary material for: Profiling the Murine Acute Phase and Inflammatory Responses to African Snake Venom: An Approach to Inform Acute Snakebite Pathology
Source: Toxins (Basel). 2022 Mar 22;14(4):229. doi: 10.3390/toxins14040229 (PMC9028489; doi:10.3390/toxins14040229)
Supplement: Supplementary file 1 [file toxins-14-00229-s001.zip › toxins-1601964-supplementary.pdf]

Supplementary Materials: Profiling the Murine Acute Phase and Inflammatory Responses to African Snake Venom: An Approach to Inform Acute Snakebite Pathology

Jaffer Alsolaiss, Chloe A. Evans, George O. Oluoch, Nicholas R. Casewell and Robert A. Harrison

Supplementary Table S1. Prior murine *in vivo* studies of the inflammatory response following dosing with snake venom. Key: IL-1/-6/-8/-10/-12p70: Interleukin-1/-6/-8/-10/-12p70; TNF-α: Tumour Necrosis Factor-α; Interferon-γ: IFN-γ; TXB_2 /4; Thromboxane B2/4; NO: Nitric Oxide; LTB_4: Leukotriene B4; CCL2: Monocyte Chemotactic Protein-1; COX-2: Cyclooxygenase-2; PGE_2/D_2: Prostaglandin E2/D2; CysLTs: Cysteinyl Leukotriene Receptors.

Snake species	Whole venom/venom toxin	Target species (Mouse strains)	Inflammatory mediators (time post-envenomation in hours)	Observed pathologies	Reference
Bothrops asper	**Whole venom**	**Swiss mice**	**Pro-inflammatory cytokines: IL-6 (1-3), TNF-α (1-6)**<br>**Eicosanoids pathway: LTB_4 (0.5-1), TXB_2 (0.5-1)**	**Leucocyte infiltrate (neutrophils)**	[1]
	Myotoxic PLA_2, P-I type metalloproteinase	Swiss mice	**Pro-inflammatory cytokines:** IL-1 (1-6), IL-6 (1-6)	Not reported	[2]
	Whole venom, myotoxic PLA_2 isoform	Swiss mice	**Pro-inflammatory cytokines:** IL-6 (3-6)	Leucocyte infiltrate (neutrophils, mononuclear cells), reduced platelets, oedema	[3]
	Whole venom	BALB/c mice	**Pro-inflammatory cytokines:** IL-1 (2-24), IL-6 (2-6), IFN-γ, TNF-α (2-18)<br>**Anti-inflammatory cytokines:** IL-10 (4-24)<br>**Other:** NO (2-24)	Not reported	[4]
Bothrops atrox	Whole venom	Swiss mice	**Pro-inflammatory cytokines:** IL-6 (1-4), IL-12p70 (4-8), TNF-α (1)<br>**Anti-inflammatory cytokines:** IL-10 (8)<br>**Chemokines:** CCL-2 (1-8)<br>**Eicosanoid pathway:** COX-2 (1-4), LTB_4 (1), PGD_2 (1), PGE_2 (1-4), TXB_2 (1)	Vascular permeability, leucocyte infiltration (mononuclear and polymorphonuclear leucocytes)	[5]
	Whole venom	BALB/c	**Pro-inflammatory cytokines:** IL-6 (1-6), IFN-γ (1-12), TNF-α (1-18)	Leucocyte infiltration (polymorphonuclear cells), skeletal muscle	[6]

			**Anti-inflammatory cytokines:** IL-10 (1-18) **Other:** NO (1-24)	disruption, oedema, erythema, haemorrhage and local tissue necrosis	
	P-I metalloprotease, PLA_2	C57BL/6	**Pro-inflammatory cytokines:** IL-1β (4, P-I metalloprotease only), IL-6 (1-2, PLA_2; 2, P-I metalloprotease) **Anti-inflammatory cytokines:** IL-10 (1, PLA_2; 1-2, P-I metalloprotease **Eicosanoid pathway:** CysLTs (1-2, PLA_2 only), LTB_4 (4, PLA_2 only), PGE_2 (4, both toxins)	Leucocyte infiltration (neutrophils, mononuclear cells)	[7]
	Whole venom	C57BL/7	**Pro-inflammatory cytokines:** IL-1β (4.5), IL-6 (4.5) **Anti-inflammatory cytokines:** IL-10 (4.5) **Chemokines:** CCL-2 (4.5) **Eicosanoid pathway:** LTB_4 (4.5), COX-2 (4.5), TXB_2	Leucocyte infiltration (mononuclear and polymorphonuclear leucocytes)	[5]
Bothrops jararaca	Whole venom	BALB/c mice	**Pro-inflammatory cytokines:** IL-1 (4-24), IL-6 (4-18), TNF-α (2-18), IFN-γ (2-24) **Anti-inflammatory cytokines:** IL-10 (4-24 hours)	Not reported	[4,8]
Bothrops jararacaussu	Whole venom	Swiss mice	**Pro-inflammatory cytokines:** IL-1β (4.5), TNF-α (4.5) **Eicosanoid pathway:** COX-2 (4.5)	Leucocyte infiltration (neutrophils), oedema, haemorrhage, and necrosis	[9]

Supplementary Table S2. The determined median venom lethal dose 50 (LD_{50}) doses of the African snake venoms used in this study.

Snake species	LD50 doses (µg venom/mouse (18-22g) (95% confidence intervals)	Mice doses selected for analysis (µg venom/mouse (18-22g)	Number of surviving mice from each group used in this study (Overall group size, n=5)
Bitis gabonica	18.4 (17.4- 19.5)	10	3
Bitis arietans	17.7 (17.2- 18.2)	15	3
Echis leucogaster	56 (31.1- 100.5)	35	3
Echis ocellatus	58 (33.0- 101.9)	38	2
Naja haje	6.9 (5.3- 9.0)	8	2
Naja melanoleuca	14.5 (13.5- 15.5)	13	3
Naja nigricollis	27.5 (25.2- 29.9)	24	3
Dendroaspis polylepis	11.5 (10.5- 12.5)	8	3
Dendroaspis viridis	13.2 (11.7- 14.8)	10	2
Dendroaspis jamesoni	54 (46.9- 61.8)	42	2

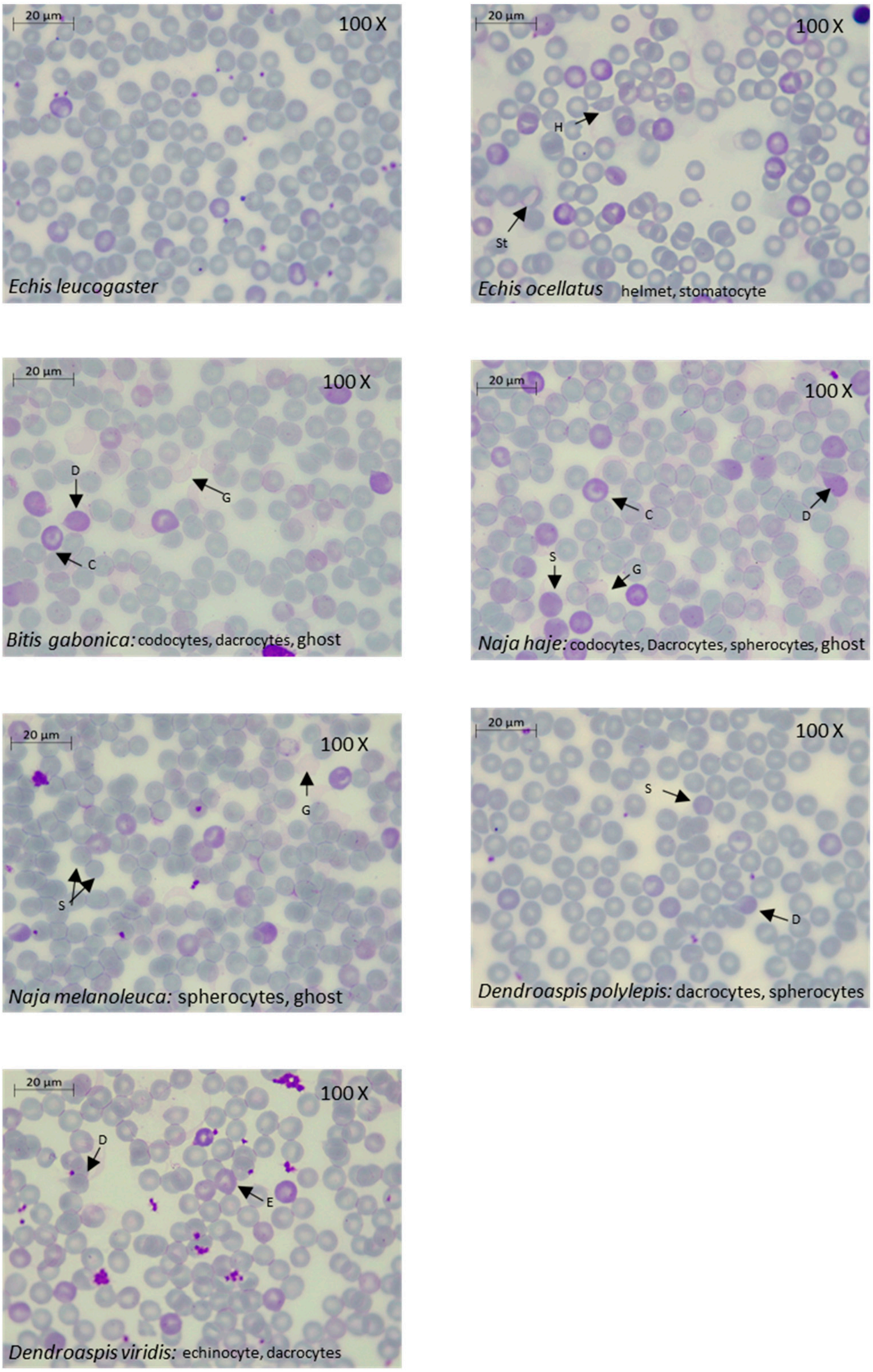

Supplementary Figure S1. Representative images of red blood cell morphology (100 × objective) observed in thin blood films of mice injected with different African snake venoms.

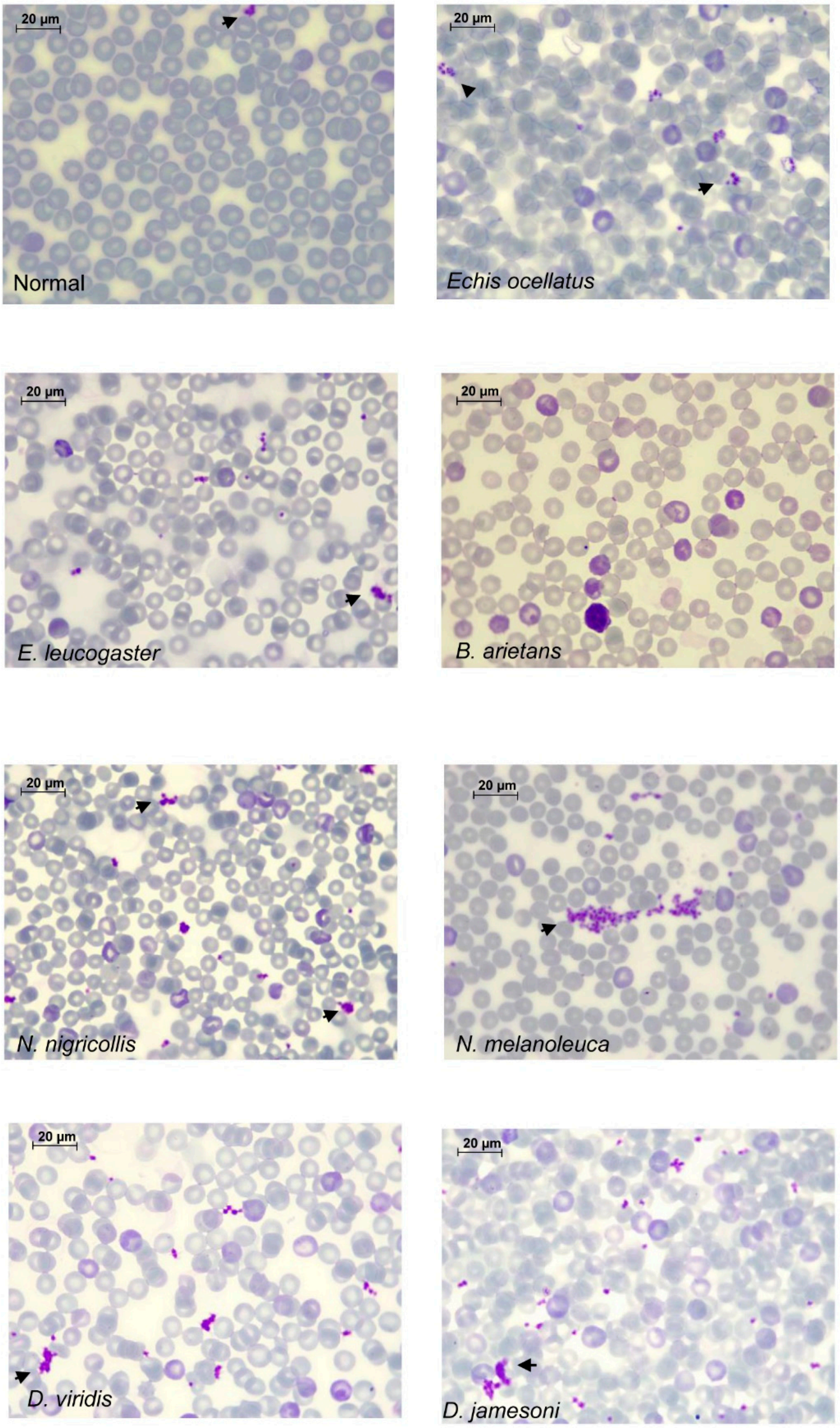

Supplementary Figure S2. Examples of platelet aggregation (100 × objective) observed in blood films of mice injected with different venoms. Arrows indicated platelet aggregation colonies compared to the normal blood film control.

1. Zamuner, S.R.; Zuliani, J.P.; Fernandes, C.M.; Gutiérrez, J.M.; de Fátima Pereira Teixeira, C. Inflammation induced by Bothrops asper venom: Release of proinflammatory cytokines and eicosanoids, and role of adhesion molecules in leukocyte infiltration. *Toxicon Off. J. Int. Soc. Toxinol.* **2005**, *46*, 806–813.
2. Rucavado, A.; Escalante, T.; Teixeira, C.F.P.; Fernandes, C.M.; Diaz, C.; Gutierrez, J.M. Increments in cytokines and matrix metalloproteinases in skeletal muscle after injection of tissue-damaging toxins from the venom of the snake Bothrops asper. *Mediat. Inflamm.* **2002**, *11*, 121–128.
3. Lomonte, B.; Tarkowski, A.; Hanson, L.A. (Eds.) *Host Response to Bothrops asper Snake Venom: Analysis of Edema Formation. Inflammatory Cells, and Cytokine Release in a Mouse Model*; Plenum Publishing Corporation: New York ,USA, 1993.
4. Petricevich, V.L.; Teixeira, C.F.P.; Tambourgi, D.V.; Gutiérrez, J.M.A. Increments in serum cytokine and nitric oxide levels in mice injected with Bothrops asper and Bothrops jararaca snake venoms. *Toxicon* **2000**, *38*, 1253–1266.
5. Moreira, V.; Dos-Santos, M.C.; Nascimento, N.G.; da Silva, H.B.; Fernandes, C.M.; Lima, M.R.D.; Teixeira, C. Local inflammatory events induced by Bothrops atrox snake venom and the release of distinct classes of inflammatory mediators. *Toxicon Off. J. Int. Soc. Toxinol.* **2012**, *60*, 12–20.
6. Barros, S.F.; Friedlanskaia, I.; Petricevich, V.L.; Kipnis, T.L. Local inflammation, lethality and cytokine release in mice injected with Bothrops atrox venom. *Mediat. Inflamm.* **1998**, *7*, 339–346.
7. Menaldo, D.L.; Bernardes, C.P.; Zoccal, K.F.; Jacob-Ferreira, A.L.; Costa, T.R.; Del Lama, M.P.F.M. Immune cells and mediators involved in the inflammatory responses induced by a P-I metalloprotease and a phospholipase A2 from Bothrops atrox venom. *Mol. Immunol.* **2017**, *85*, 238–247.
8. Clissa, P.B.; Laing, G.D.; Theakston, R.D.G.; Mota, I.; Taylor, M.J.; Moura-da-Silva, A.M. The effect of jararhagin, a metalloproteinase from Bothrops jararaca venom, on pro-inflammatory cytokines released by murine peritoneal adherent cells. *Toxicon* **2001**, *39*, 1567–1573.
9. Wanderley, C.; Silva, C.; Wong, D.; Ximenes, R.; Morelo, D.; Cosker, F.; Aragão, K.; Fernandes, C.; Palheta-Júnior, R.; Havt, A.; et al. Bothrops jararacussu snake venom-induces a local inflammatory response in a prostanoid- and neutrophil-dependent manner. *Toxicon Off. J. Int. Soc. Toxinol.* **2014**, *90*, 134–147.
